# Application of convolutional neural networks for classification of adult mosquitoes in the field

**DOI:** 10.1371/journal.pone.0210829

**Published:** 2019-01-14

**Authors:** Daniel Motta, Alex Álisson Bandeira Santos, Ingrid Winkler, Bruna Aparecida Souza Machado, Daniel André Dias Imperial Pereira, Alexandre Morais Cavalcanti, Eduardo Oyama Lins Fonseca, Frank Kirchner, Roberto Badaró

**Affiliations:** 1 University Center SENAI CIMATEC, National Service of Industrial Learning–SENAI, Salvador, Bahia, Brazil; 2 Health Institute of Technologies (CIMATEC ITS), National Service of Industrial Learning–SENAI, Salvador, Bahia, Brazil; 3 Research Centre for Artificial Intelligence, DFKI, Bremen, Germany; Instituto Nacional de Salud Pública, MEXICO

## Abstract

Dengue, chikungunya and Zika are arboviruses transmitted by mosquitos of the genus *Aedes* and have caused several outbreaks in world over the past ten years. Morphological identification of mosquitos is currently restricted due to the small number of adequately trained professionals. We implemented a computational model based on a convolutional neural network (CNN) to extract features from mosquito images to identify adult mosquitoes from the species *Aedes aegypti*, *Aedes albopictus* and *Culex quinquefasciatus*. To train the CNN to perform automatic morphological classification of mosquitoes, we used a dataset that included 4,056 mosquito images. Three neural networks, including LeNet, AlexNet and GoogleNet, were used. During the validation phase, the accuracy of the mosquito classification was 57.5% using LeNet, 74.7% using AlexNet and 83.9% using GoogleNet. During the testing phase, the best result (76.2%) was obtained using GoogleNet; results of 52.4% and 51.2% were obtained using LeNet and AlexNet, respectively. Significantly, accuracies of 100% and 90% were achieved for the classification of *Aedes* and *Culex*, respectively. A classification accuracy of 82% was achieved for *Aedes* females. Our results provide information that is fundamental for the automatic morphological classification of adult mosquito species in field. The use of CNN's is an important method for autonomous identification and is a valuable and accessible resource for health workers and taxonomists for the identification of some insects that can transmit infectious agents to humans.

## Introduction

Arthropod-borne viruses are responsible for more than 100 of the diseases that comprise the estimated global burden of communicable human diseases[1]. Vector-borne diseases cause more than one billion infections and more than one million deaths in humans every year[2]. Dengue, chikungunya and Zika are the most common arboviruses and have caused several major epidemics in France Polynesia and Latin America during the past 10 years[3–6]. These three viral diseases are among the greatest public health challenges in the world[7]. Entomology research is considered to be a priority by the World Health Organization to develop tools that can be applied to reduce incidence and mortality and prevent epidemics caused by vector-borne diseases worldwide[2].

*Aedes aegypti* and *Aedes albopictus* have received worldwide attention since both species are efficient vectors for the transmission of human arboviral diseases, such as Zika, dengue, chikungunya, and yellow fever[3,8,9]. Additionally, controversial studies have also indicated that the genus *Culex* could be a possible vector of the Zika virus[10–12]. *Ae*. *aegypti*, *Ae*. *albopictus* and *C*. *quinquefasciatus* are very common domiciliary mosquito vectors that are present in almost all urban areas in Brazil[7]. *Ae*. *aegypti*, *Ae*. *Albopictus* are, among all *Aedes* species, the ones that circulate in endemic areas. Furthermore, *Aedes* mosquitoes have been demonstrated to be capable of transmitting Zika and other viruses in many tropical areas in the Americas, Africa, and Asia[13].

Entomological characterization is fundamental for acquiring information about mosquito behaviour. In general, the current procedure used to identify the species of an insect requires an individual visual examination of the insect, which is time consuming and requires several years of experience[14,15]. While the general interest in documenting the diversity of insect species has grown exponentially over the years, the number of taxonomists and other professionals trained in species identification has steadily declined[16–18]. Important factors that can impede the correct identification of mosquito species include the method of preservation used during the transport of samples and the use of the appropriate equipment to capture mosquitoes without damaging them[19]. Due to that, developing a tool to allow the classification of adult mosquitoes in the field, considering the environmental issues, would be very important[20].

Another possibility for species identification is the use of molecular techniques that have been validated by various studies, such DNA barcoding, environmental DNA testing and real-time PCR (qPCR)[21–23]. However, molecular identification of mosquitoes is a slow and expensive process for most laboratories.

Recently, new models that facilitate the automatic classification of mosquitoes have been developed. Some studies have attempted to classify mosquito species based on the frequency and harmonics of their wingbeats[24,25]. Techniques based on image feature analysis have also been used as a classification method [26–29]. In addition, Machine Learning and Deep Learning techniques have been used for mosquito classification[30–32]. Most of these studies, however, aimed to develop methods and tools that requires a laboratory environment. The development of a tool to support taxonomist and health workers in the field would be very helpful in accelerating the knowledge of which mosquito is circulating in the community and supporting health authorities in controlling harmful mosquitoes, once it will reduce the lag between the time the trap is placed and the taxonomic inspection occurs[33].

Wingbeat vibration techniques have been reported to demonstrate a high accuracy in the classification of mosquitoes[32]. However, the sensors used have a limited amount of memory and are unable to store the entire data stream for later processing. Indeed, the sensors must process the data stream in real time to identify events of interest and filter out background noise[34].

Within the field of image feature analysis, studies have been conducted to identify mosquitoes according to their life cycle stage (egg, larval phase, pupal stage and adult). Some studies have attempted to automatize the process of egg counting to assess the level of fecundity and thereby estimate the mosquito population size and conduct morphometric analysis[35–38]. Studies conducted to classify mosquito-based image in adult phase still focus on parts of the body of the mosquito. The outlines of body parts, such as wings, are stable and diverse but have not been frequently used in conventional taxonomy due to difficulties with lexical descriptions[15]. A classification method capable of analysing the features of the whole body is essential, since in the field there is no equipment that allow the health worker to separate parts of the body and analyse it.

Deep learning methods are essential for the processes underlying general object recognition[39]. In 2012, during the ImageNet Large Scale Visual Recognition Challenge (ILSVRC), which was the largest contest conducted in the object recognition field, a breakthrough in deep learning occurred when a convolutional network won the competition by reducing the state-of-the-art top-5 error rate from 26.1% to 15.3%[40]. Convolutional neural networks (CNN) constitute a class of models that utilize prior knowledge to compensate for data that is not available[39]. A CNN method has been developed to classify *Aedes* mosquito larva using a small dataset; when 200 epochs were used, the network achieved 96.8% accuracy[31].

It should be emphasized that correct identification of adult *Aedes* mosquito species is essential for both the recognition of the vectors involved in disease transmission and the development of fast and efficient control strategies[15]. In this study, we compared three CNN's, LeNet, AlexNet and GoogLeNet to evaluate the potential application of a Deep Learning technique to classify adult mosquito species in the field. The objective is to develop an epidemiological tool to be used in the real-world environment to facilitate the work of entomologists and health workers in the classification of adult mosquitoes of the species *Ae*. *aegypti*, *Ae*. *albopictus* and *C*. *quinquefasciatus*. As showed, this tool would be very useful to reduce the time, to allow its use by less experienced expert and to capture preserved features of the body characteristics of the mosquito. Another objective of this work is to initiate a preliminary scientific study that would allow the community be part of the control of vector-borne diseases.

Considering that the selection of algorithm training parameters is essential to improve the accuracy and reliability of the method, this study also applied the statistical analysis prior to the selection to determine the parameters set to be used to classify the mosquitoes.

## Materials and methods

### Ethics statement

No permits were required for sampling for this study. The field sampling did not involve any endangered or protected species.

### Sample collection

The mosquito samples used for image capture were obtained from the Parasitology Laboratory of the Federal University of Bahia–UFBA (Salvador, Brazil) and were also collected in the field. The samples obtained from UFBA were *Ae*. *aegypti* mosquitos (10 females and 6 males). The capture of adult insects in the field resulted in the collection of 73 specimens of *Ae*. *aegypti*, 94 specimens of *Ae*. *albopictus* and 110 specimens of *C*. *quinquefasciatus*.

The field sampling took place between September and October 2017 in the city of Salvador in two collection areas (Bahia, Brazil). CDC light traps and suction tubes were used for the collection of adult insects.

The captured specimens were euthanized with ethyl acetate and stored in entomological collection tubes until identification was performed by an entomologist.

### Construction of the dataset: Structure, acquisition and distribution

A robust, correctly structured dataset was created to allow the differentiation of the *Ae*. *aegypti*, *Ae*. *albopictus* and *C*. *quinquefasciatus* species. In addition, differentiation of males and females was conducted as described in a previous study[41]. A model was developed to classify the mosquitoes into six different classes according to gender and species. At this stage, our work does not intend to classify other species and non-mosquitoes images.

The images in the dataset were extracted from the ImageNet platform and were also photographed with various cameras, including a Leica DMC2900 (Leica Microsystems, Heerbrugg, Switzerland) coupled to a stereoscopic Leica M205C at the Oswaldo Cruz Institute of Entomology at FIOCRUZ (Rio de Janeiro–Brazil), a Canon Power Shot D30 (Canon, Tokyo, Japan) coupled to a Wild M3C stereomicroscope (Leica Microsystems, Heerbrugg, Switzerland) at SENAI CIMATEC (Salvador–Brazil) and Samsung J5 (Samsung, Seoul, South Korea) and Apple iPhone 7 (Apple, Cupertino, California, USA) mobile phone cameras. The images were collected at different resolutions and levels of quality to develop a classification method that utilized a wide range of images to prevent possible overfitting of the trained model. An average of 10 photographs of each specimen were taken at different angles and proximities.

**Fig 1:**
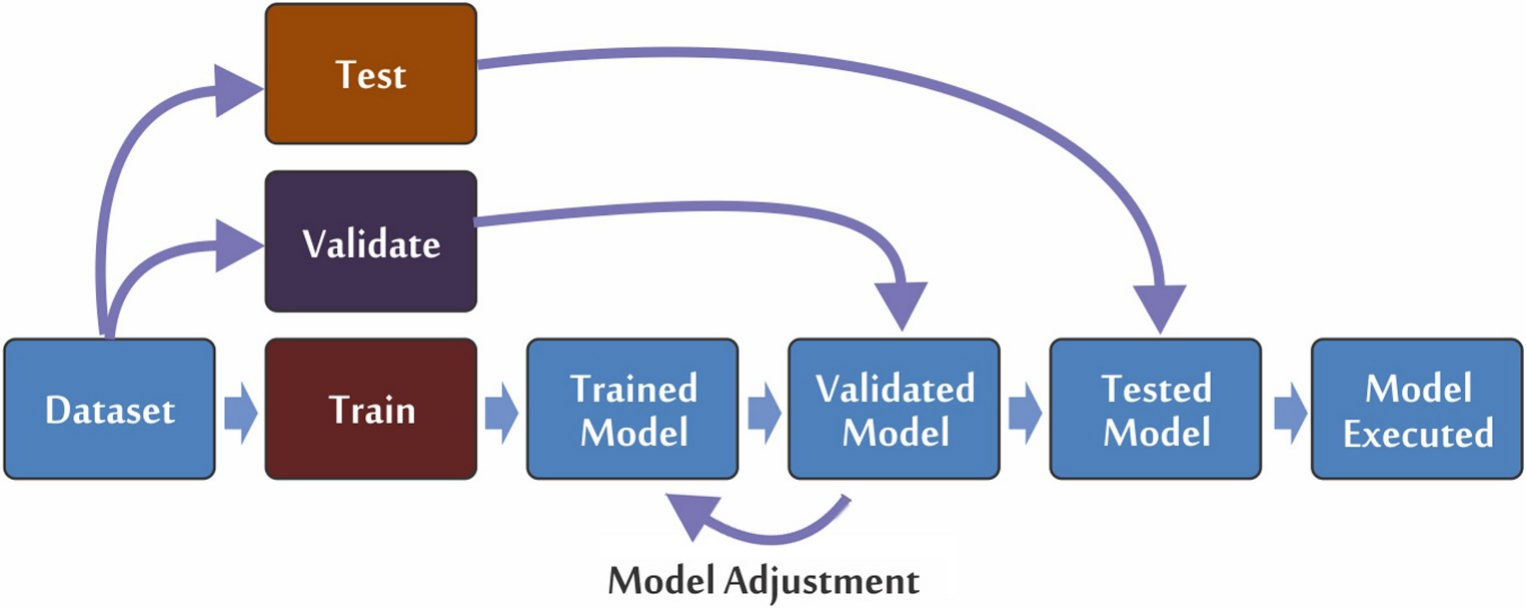
Representation of the training (phase 1), validating (phase 2) and testing (phase 3) processes of the mosquito classification model.

Once the dataset was structured and the images acquired, the number of images to be used for training, validating and testing the model was determined. Fig 1 summarizes how the dataset was used in the computational model and shows the three different phases of the development of the model: training (phase 1), validation (phase 2) and testing (phase 3).

### Determination of the application images

The application images were those that we intended use for the classification of mosquitoes after the model was developed. This was done to allow the model to be used by any person, not only a specialist using a microscope, who is able to photograph an insect and then run the model to classify mosquitoes.

**Fig 2:**
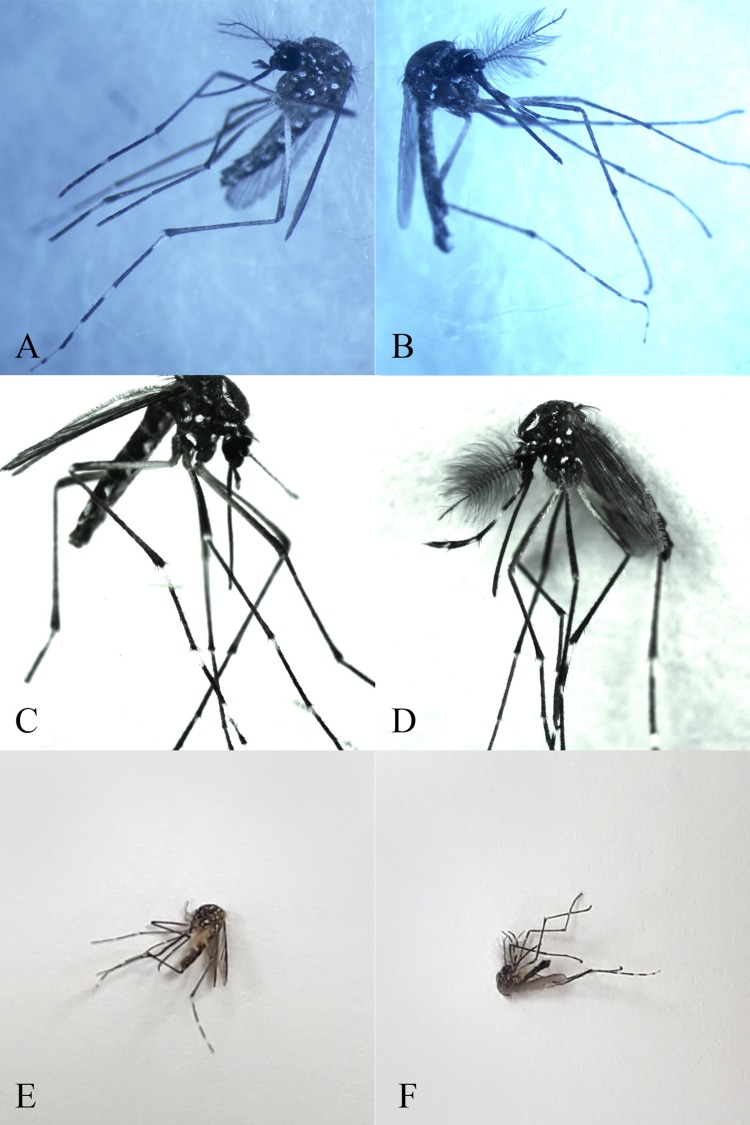
Images used for the validation phase during the development of the model used in this study. (A and C) *Ae*. *aegypti* females photographed using a digital camera; (B and D) *Ae*. *aegypti* males photographed using a digital camera; (E) an *Ae*. *aegypti* female photographed using a mobile phone; (F) an *Ae*. *aegypti* male photographed using a mobile phone.

A total of 823 out of the 4,056 images used in this study were manually selected according to the following characteristics: the image should show the entire insect body (to visualize all relevant morphological features) and should have been taken with a mobile phone. Fig 2 shows some captured images of male and female *Ae*. *aegypti* photographed using a digital camera (Fig 2A, 2B, 2C and 2D) and mobile phone (Fig 2E and 2F).

### Determination of the application images to be used for testing

For the testing phase (phase 3), images were extracted from the dataset and used only after the model was validated. Additionally, the images used for testing were not used to train or validate the model. This was done to avoid research bias and minimize the chance of overfitting.

To standardize the analysis of the results, the test dataset contained the same number of images from each of the six classes of mosquitoes. It was decided that the number of images corresponding to 20% of the number of images in the smallest class would be used for testing for all classes. As the *C*. *quinquefasciatus* female class was the class with the smallest number of application images (70 images), fourteen images (20%) from each of the other classes were randomly removed from the dataset. Python scripts were employed to randomize the chosen images.

### Distribution of the application images used for the training and validation phases

Within the references used in this work, no consensus was found concerning the ideal dataset distribution between the training (phase 1), validation (phase 2) and testing (phase 3) phases.

To determine the best image distribution for training and validation, once the test sample size was already defined, the percentage of application images used in the training phase was defined as a factor, varying in four levels (either 30%, 40%, 50% or 60%), in a full factorial experiment. The objective was to evaluate statistically the best distribution of application images between the training and validation phases that would present the best classification accuracy for the intended application in entomology.

### Selection of neural networks

We used nVidia DIGITS software (nVidia, Santa Clara, California, USA) with a NVIDIA GeForce GTX TITAN GPU for processing. We used the software on the Linux Ubuntu operating system LTS Distribution 16.04 (Linux, San Francisco, California, USA). The Caffe framework was used for the LeNet[42,43], AlexNet[31] and GoogLeNet convolutional neural networks [42,43].

For a long period, LeNet was considered the state of the art of Artificial Neural Networks (ANN's). LeNet was one of the first ANN's used to improve the original backpropagation algorithm; the remarkable development of this well-known network was the first step towards the application of the Deep Learning method.

In 2012, Alex Krizhevsky released AlexNet, which was a deeper and much wider version of the LeNet that won, by a large margin, the difficult ImageNet competition[39]. AlexNet has a very similar architecture to that of LeNet, but it can better describe images. This network relies on eight layers, including convolutional, local responses, max-pooling and fully connected layers.

In 2014, Christian Szegedy, from Google, began to investigate methods to reduce the computational burden of deep neural networks[43]. In doing so, he focused on the efficiency of the architecture of the deep neural network (codenamed *Inception*, later named *GoogLeNet)*. This neural network was the winner of ILSVRC 2014, surpassing AlexNet, and was proclaimed the new paradigm for convolutional neural networks.

### Classification of the model parameters

The CNN algorithms requires the definition of several parameters prior to the training phase. Depending on the application, these parameters might have more or less influence on the classification results. Prior random tests were performed to understand which set parameters have more influence and should be statistically analysed as a factor in the full factorial experiment in order to stablish the best value. Other parameters with less influence on the results were set as default in the algorithm.

Number of epochs and Seed were parameters defined as a constant value. The prior random tests showed that 200 epochs are enough to stabilize the training and validation phases. In case it generates overfitting it would be easily identified. Define the seed as a constant value was important to standardize the neural network weights initiation for all CNN and also, for each time we run the algorithm. The parameters evaluated in the full factorial experiment were: percentage of the application image used in the training phase; solver algorithm; the learning rate (LR); and learning rate decay function. The other parameters, such as the batch size, batch accumulation, learning rate step size, learning rate power, learning rate gamma, crop size and mean subtraction, were set according to pre-defined default for each network.

### Full factorial experiments

To understand the influence of each parameter and their correlations with one another, full factorial experiments were conducted using the Minitab 17 software (Minitab, State College, Pennsylvania, USA). The parameters used as the inputs for the full factorial experiments are shown in Table 1.

**Table 1:** Parameters used as inputs for the full factorial experiments in this study.

Parameters	Levels	Level			
		1	2	3	4
Number of epochs	1	200			
Seed	1	10			
% application images used at training	4	30	40	50	60
Solver algorithm	4	SGD	NAG	Adam	AdaGrad
Learning rate	3	0.001	0.01	0.1	
LR decay function	4	Step Down	Exponential	Sigmoid	Polynomial

Using the Minitab software, we created an experiment with four factors: the percentage of application images used at training, solver algorithm, learning rate, and LR decay function. The number of epochs and the seed was fixed. For the experiments, we defined the number of levels as shown in Table 1. During the next step, the formats of the selected parameters were defined as either numerical or text; the percentage of application images used at training and the LR were defined as numerical, and the solver algorithm and LR decay function were defined as text. The values for each level was defined based on the random experiments performed before.

Based on the result of the factorial experiments, it was calculated that 192 experiments should be conducted on one of the three convolutional neural networks. The output variables to be evaluated were the percent accuracy during the validation phase, loss function and percent accuracy during the testing phase.

### Full factorial experiments analysis

Based on the full factorial experiments, GoogLeNet was the network selected for conducting the 192 planned experiments.

The first step in the analysis was to interpret the statistical significance of the parameters that were used (percent accuracy during the validation phase, loss function and percent accuracy during the testing phase) and their relationship with the results. The ‘P value’ was calculated to evaluate the statistical significance of the parameters and the effects of their interactions on the outcome of the experiments.

The best performance obtained for each outcome was then defined as the target value for optimization analysis and was used to determine the best combination of parameters. These combinations were used to train the other two convolutional neural networks, LeNet and AlexNet.

## Results

The species and gender of each of the dataset classes used to classify the 4,056 images are shown in Table 2.

**Table 2:** Dataset structure and sample sizes of the mosquito images used in this study.

Class name	Total number of images	Non-application images (used in training)	Application images	Application images used for training and validation	Application images used for testing
*Ae*. *aegypti* female	947	723	224	210	14
*Ae*. *aegypti* male	282	201	81	67	14
*Ae*. *albopictus* female	1050	821	229	215	14
*Ae*. *albopictus* male	436	327	109	95	14
*C*. *quinquefasciatus* female	965	895	70	56	14
*C*. *quinquefasciatus* male	376	266	110	96	14

**Fig 3:**
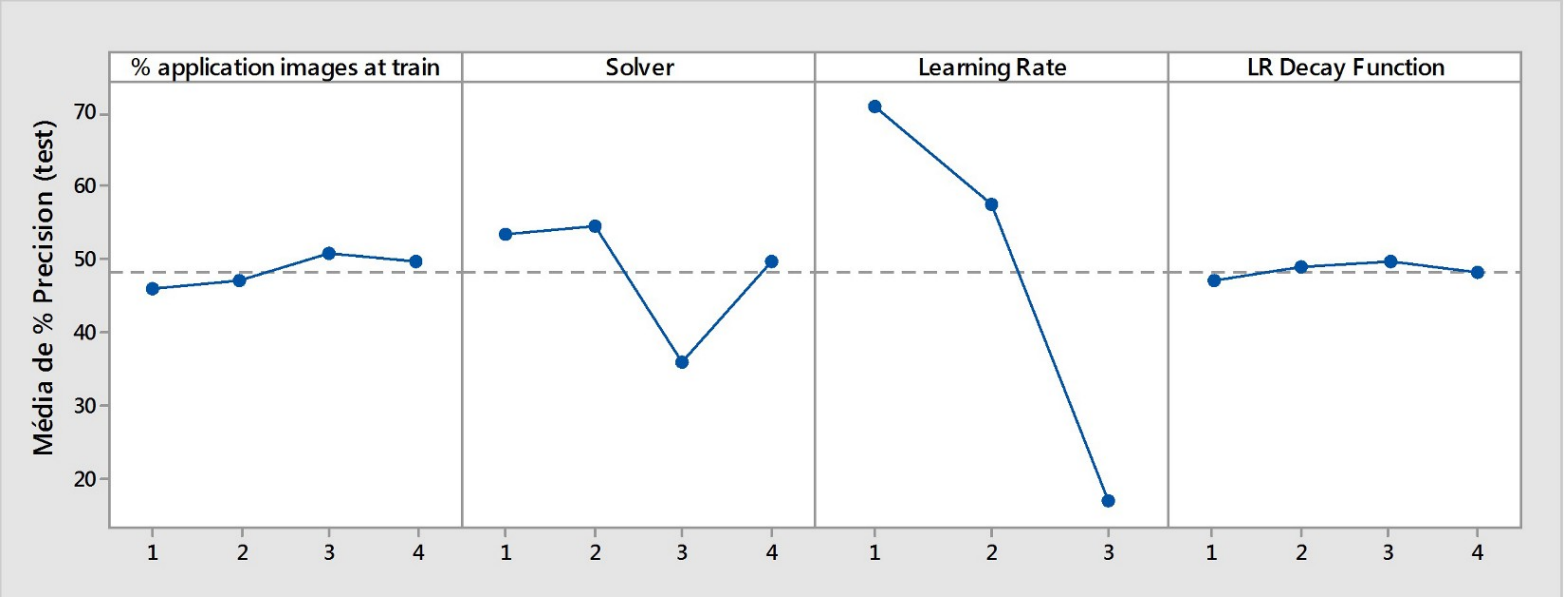
Effects of the first level parameters on the accuracy of the testing phase (average accuracy percentage).

Fig 3 shows the effects of each parameter (1^st^ level analysis) on the accuracy of the testing phase (phase 3). In this analysis, the use of the solver algorithm ‘3’ (Adam) and a learning rate of ‘3’ (0.1) significantly reduced the accuracy of the mosquito image classification. However, a learning rate of ‘1’ (0.001) was an important parameter that was used (‘p value’ < 0.00001). Almost every parameter had a significant influence on the results of the performance of the trained neural network (‘p value’ < 0.05), with the exception of the interaction between ‘% application images (train) * Solver’ and ‘% application images (train) * LR Decay Function’ (‘p value’ > 0.05).

**Fig 4:**
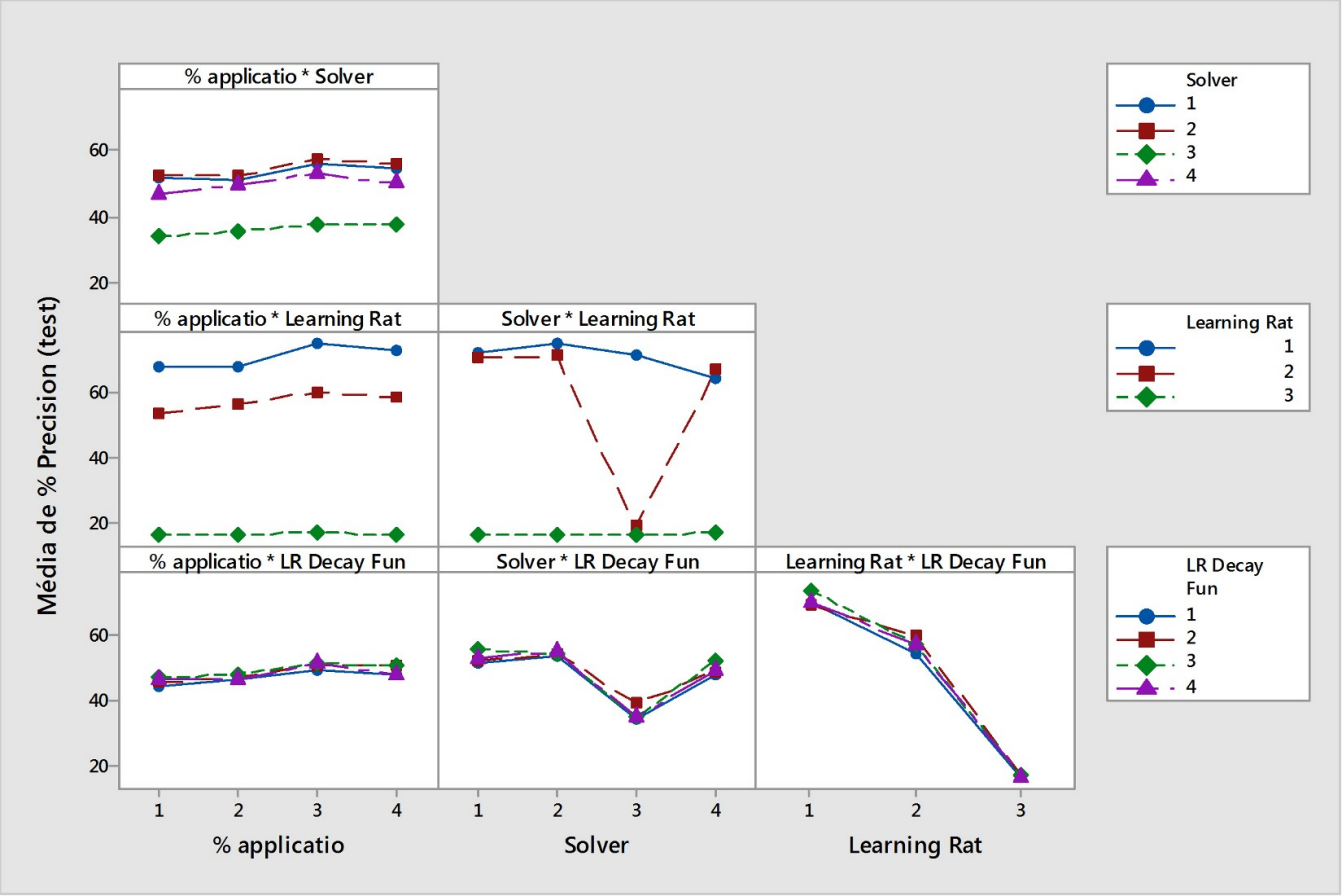
Effects of the second level parameters on the accuracy of the testing phase (average accuracy percentage).

Fig 4 shows the effects of the parameter combination (2^nd^ level analysis) on the interaction of the parameters with one another. It is clear that a learning rate of ‘3’ (0.1) reduced the accuracy of the testing phase when combined with any of the other parameters. Consequently, this learning rate should not be used subsequent analysis. It was also confirmed that the solver algorithm ‘3’ (Adam) reduced the accuracy of the testing phase. However, the combination of a learning rate of ‘2’ (0.01) with solver algorithm ‘4’ (AdaGrad) obtained better results than the combination of a learning rate of ‘1’ (0.001) with the same solver algorithm (‘p value’ < 0.00001). Overall, the learning rate of ‘1’ (0.001) produced the best results.

**Fig 5:**
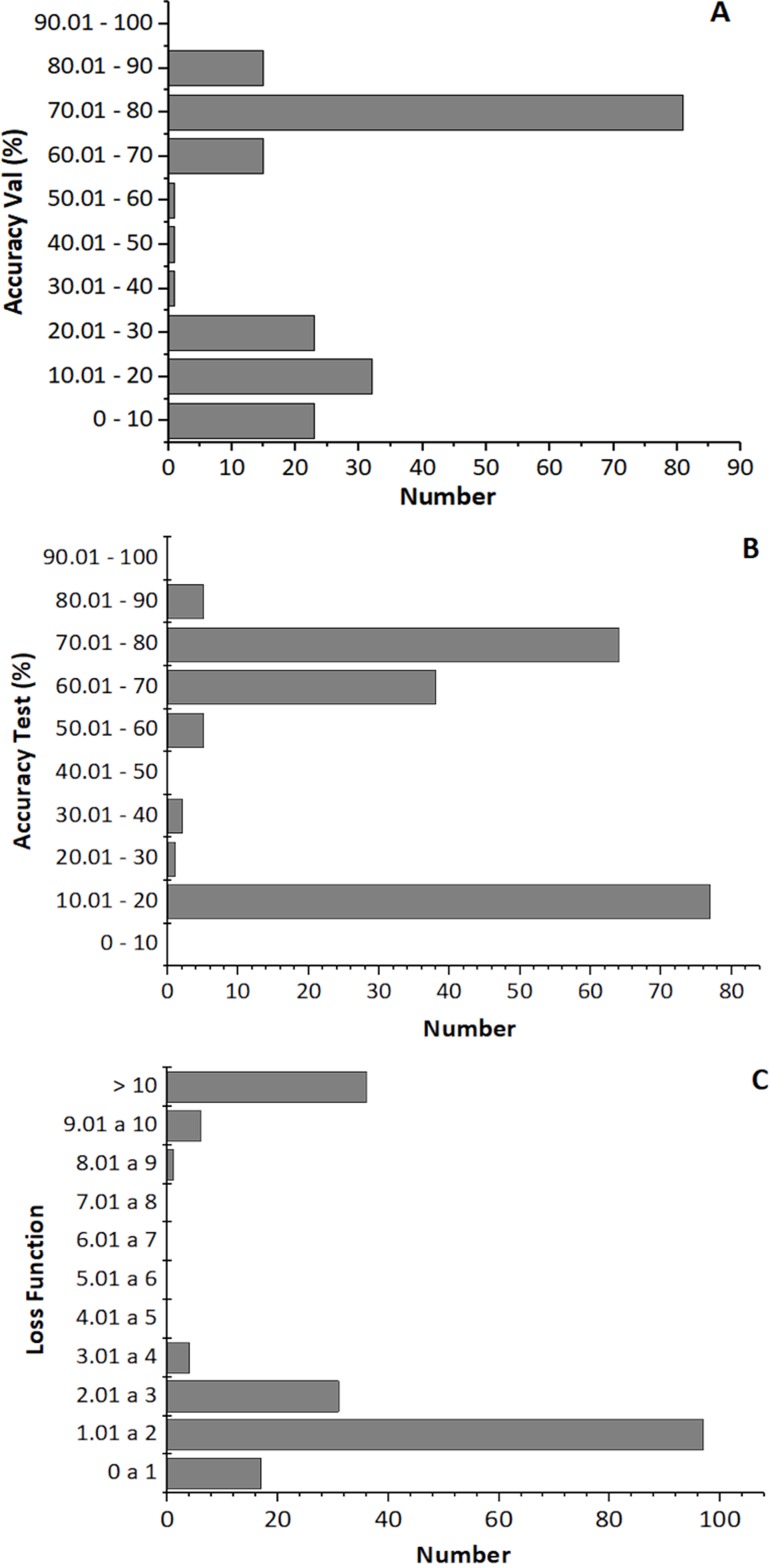
**Histograms representing the outcome data**: (A) percent accuracy–validation, (B) percent accuracy–testing, (C) loss function.

To define the best set of parameters, an optimization function was calculated in Minitab that set the target values for each outcome. Fig 5 shows the variation of the data obtained for each outcome. The best performance in terms of the percentage accuracy during validation was 83.9%, with a loss function of 0.67 and percentage accuracy during testing of 86.9%.

The best parameter combination was obtained in the 4-2-1-3 set, which resulted in the use of 60% of the application images during training (the NAG Solver algorithm; 0.001 LR and Sigmoid LR decay function). Therefore, this parameter set was used for the subsequent training of all three convolutional neural networks.

**Fig 6:**
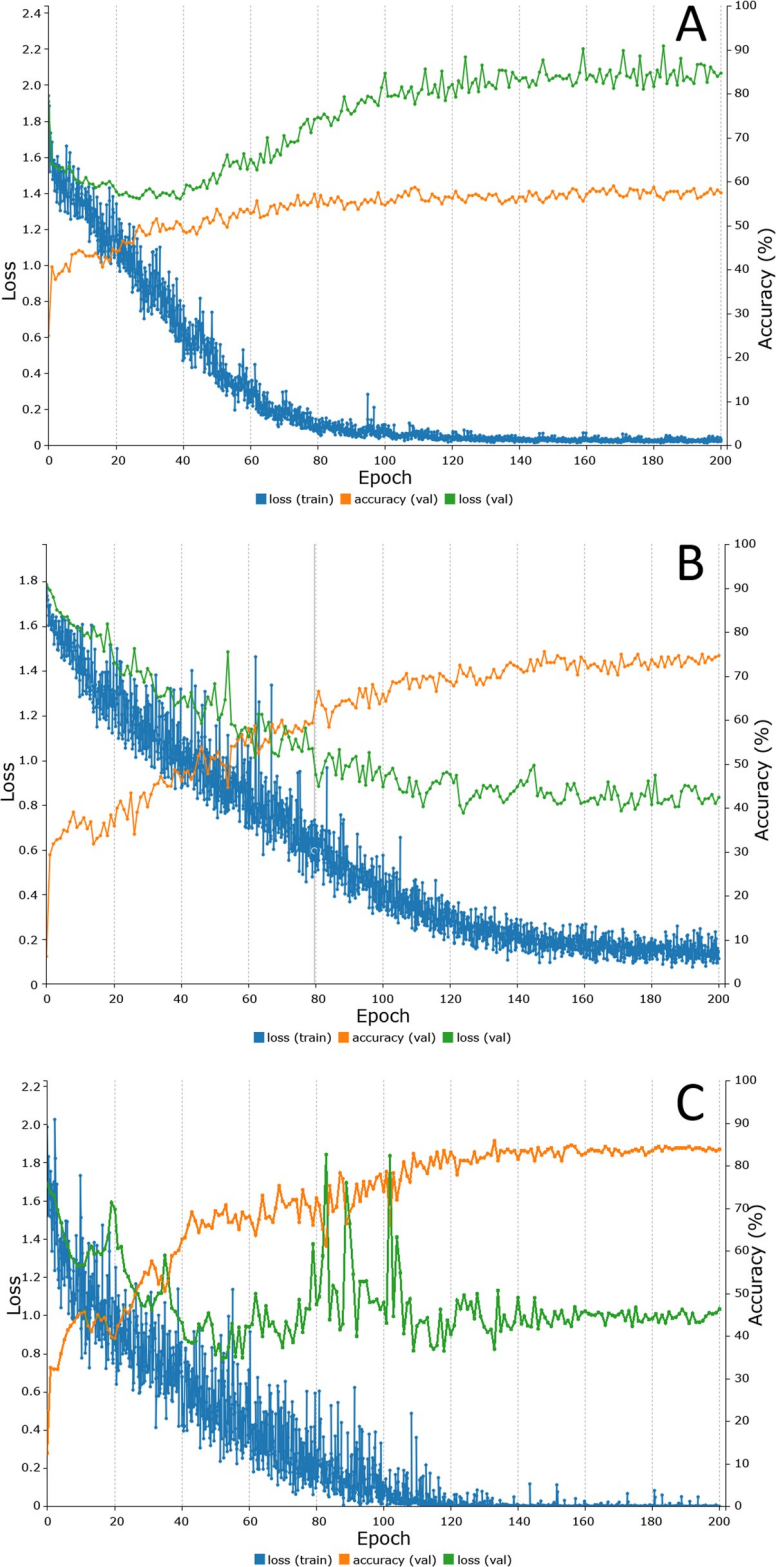
**Representation of the training process of each CNN: (A) LeNet, (B) AlexNet and (C) GoogLeNet (orange line: accuracy validation; green line: loss validation; blue line: loss training).** A–LeNet (after 200 epochs): percent accuracy (validation) 57.50%, loss (validation) 2.07, loss (training) 0.03; B–AlexNet (after 200 epochs): percent accuracy (validation) 74.69%, loss (validation) 0.83, loss (training) 0.11; C–GoogLeNet (after 200 epochs): percent accuracy (validation) 83.88%, loss (validation) 1.03, loss (training) 0.00.

Fig 6 shows the results obtained during the training and validation phases for the convolutional neural networks LeNet, AlexNet and GoogLeNet.

The LeNet neural network achieved an accuracy of 57.5% for the classification of mosquitoes (Fig 6A). Interestingly, the loss function (2.07) significantly increased during the training of the network, indicating possible overfitting. Table 3 presents the accuracy achieved by this network for each mosquito species. The overall performance of the LeNet neural network during the testing phase (phase 3) was 52.4%.

**Table 3:** Confusion matrix showing the results of the testing phase for the LeNet neural network.

Class	*Ae*. *aegypti* female	*Ae*. *aegypti* male	*Ae*. *albopictus* female	*Ae*. *albopictus* male	*C*. *quinquefasciatus* female	*C*. *quinquefasciatus* male	Accuracy (%)
***Ae*. *aegypti* female**	7	1	2	4	0	0	50.00
***Ae*. *aegypti* male**	5	6	3	0	0	0	42.86
***Ae*. *albopictus* female**	1	2	10	0	1	0	71.43
***Ae*. *albopictus* male**	2	0	0	7	3	2	50.00
***C*. *quinquefasciatus* female**	1	0	1	1	9	2	64.29
***C*. *quinquefasciatus* male**	2	3	2	0	2	5	35.71

Fig 6B shows the results obtained during the training and validation phases for the AlexNet neural network. Using the previously selected parameters (200 epochs; seed of 10; 60% of application images used for training; NAG solver algorithm; 0.001 LR; sigmoid LR decay function), the network achieved an accuracy of 74.7%, and the validation and training losses were reduced by approximately 0.83 and 0.11, respectively. Table 4 presents the overall accuracy obtained during the testing phase (phase 3) for each of the mosquito species for the AlexNet neural network. The overall result of the testing phase was 51.2%.

**Table 4:** Confusion matrix showing the results of the testing phase for the AlexNet neural network.

Class	*Ae*. *aegypti* female	*Ae*. *aegypti* male	*Ae*. *albopictus* female	*Ae*. *albopictus* male	*C*. *quinquefasciatus* female	*C*. *quinquefasciatus* male	Accuracy (%)
***Ae*. *aegypti* female**	5	1	6	0	1	1	35.71
***Ae*. *aegypti* male**	2	7	2	1	0	2	50.00
***Ae*. *albopictus* female**	1	1	11	1	0	0	78.57
***Ae*. *albopictus* male**	0	2	3	9	0	0	64.29
***C*. *quinquefasciatus* female**	2	4	0	0	2	6	14.29
***C*. *quinquefasciatus* male**	2	1	0	0	2	9	64.29

Fig 6C shows the results obtained during training and validation phases for the GoogLeNet convolutional neural network. Using the previously selected parameters, the network achieved an accuracy of 83.9%, and the validation and training losses were reduced by approximately 1.03 and 0, respectively. Table 5 presents the accuracy of the results obtained by the GoogLeNet network during the testing phase for each of the mosquito species (phase 3). Overall, the performance of the GoogLeNet neural network during the testing phase was 76.2%.

**Table 5:** Confusion matrix showing the results of the testing phase for the GoogLeNet neural network.

Class	*Ae*. *aegypti* female	*Ae*. *aegypti* male	*Ae*. *albopictus* female	*Ae*. *albopictus* male	*C*. *quinquefasciatus* female	*C*. *quinquefasciatus* male	Accuracy (%)
***Ae*. *aegypti* female**	9	3	2	0	0	0	64.29
***Ae*. *aegypti* male**	1	12	1	0	0	0	85.71
***Ae*. *albopictus* female**	0	1	12	1	0	0	85.71
***Ae*. *albopictus* male**	0	1	0	13	0	0	92.86
***C*. *quinquefasciatus* female**	1	1	0	0	8	4	57.14
***C*. *quinquefasciatus* male**	0	0	0	1	3	10	71.43

With the exception of its performance during the classification of *C*. *quinquefasciatus* in the validation phase in terms of the loss function after 200 epochs, GoogLeNet demonstrated the best performance for the classification of adult mosquitoes.

It is also relevant to evaluate the classification performance for the different genera of mosquitoes. For the classification of the *Aedes* and *Culex* genera, an accuracy of 96.4% was obtained. Another important outcome was the performance in the classification of *Aedes* females, during which an overall accuracy of 82.1% was achieved.

## Discussion

To the best of our knowledge, this is the first study that uses convolutional neural networks (CNN's) to extract features from images of adult mosquitoes to identify *Ae*. *aegypti* and *Ae*. *albopictus* species in the field. Members of the genus *Aedes* are well-known carriers and disseminators of arboviruses[1,13,44,45]. Also, we included the *C*. *quinquefasciatus* mosquito in our study because its medical importance as a vector for some arboviruses associated with the recent explosive epidemic occurred in Brazil[10,12]. However, the competence of *Culex sp*. to transmit Zika virus has not been confirmed by recent studies[11,46].

Sanchez-Ortiz et al.[31] used a very similar CNN-like method as our study, but focused on the use of digital images of larva as input for a machine learning algorithm for *Aedes* larva identification. The algorithm showed excellent performance, with 100% accuracy, in the identification of *Aedes* larva; however, for other mosquito species, the misclassification rate was 30%. Additionally, the sample size used in this study was tiny. Although their review have a significant scientific contribution, it requires the use of laboratory equipment and do not address issues such as time and logistics required in the traditional classification method for capturing and analysing the samples.

In our work, we used over 4,000 mosquito images in this preliminary study of the use of CNN's for the identification of mosquitoes; with such a small sample size, we achieved an accuracy of 76.2% in the automatic recognition of species and gender for six classes of mosquito. However, it is very promising that we were able to distinguish *Aedes* mosquitoes from other mosquitoes correctly. Our method was able to achieve 100% accuracy in classifying between *Aedes* and *Culex*, although *Culex* was misclassified as *Aedes* 10% of the time during the test phase. In the recognition of the *Aedes* female, which is the most important vector of arboviruses, we achieved 82% accuracy. It is essential to understand that it is a training process and every time we add a new species further training of the network will be necessary.

The importance of insects to transmit human diseases have motivated researchers to develop an arsenal of mechanical, chemical, biological and educational tools to help mitigate the harmful effects of insects. Batista et al.[33] reinforce that the efficiency of such systems depends on the knowledge of the spatiotemporal dynamics of insects. In that work, the authors developed a sensor that can measure the wingbeat frequency of flying insects at a distance. However, the study collected data under laboratory conditions. Thus, future works would be required to obtain additional features for real-world deployment.

Taxonomists have been searching for efficient methods to meet real world insect identification requirements[47]. The correct identification of insects is critically important in entomological research, pest control, and insect-resource utilization for the development of effective control strategies for diseases transmitted by these vectors and can aid in preventing arbovirus infections in humans[2,15]. Also, identifying the vectors associated with pathogens is fundamental to understand the transmission of arthropod-borne viruses[48]. This problem is recognized since the end of the last century, and computer-based recognition systems have been developed to identify insect species automatically, not only for mosquitoes, but also for wasps, moths, spiders, bees and others[15].

Unfortunately, as mentioned previously, taxonomic identification of mosquitoes is a time-consuming and challenging process that requires trained specialists. The high variability also compromises this method of identification of the morphological and molecular characteristics found in members of the *Culicidae* subfamily, which was the object of our study. Furthermore, molecular identification, although generally accurate, is costly and often impractical. Other disadvantages of molecular techniques are the high cost and time applied in the laboratory. In this way, developing countries, which are often the main endemic areas, do not have sufficient resources for investments in laboratories with high technologies.

Although there is progress in autonomous identification systems for insects, two inadequacies remain. First, some systems obtain high accuracy of identification for limited insect species but have very narrow functionality, because the source code for those computer programs must be rewritten when these types of system are tested with new morphological insect data. Second, some systems, can be used to identify different taxonomic groups of insects, but the accuracy is not sufficient due to high variations during processes of digitizing morphological features and insufficient powerful classifiers which only can search local optimizing solutions rather than the global optimizing solutions during computation[15].

Autonomous identification method could be valuable and more accessible to health workers and other non-taxonomists for use in the identification of insects that can transmit infectious agents to humans. Herein, we demonstrated that it is possible to use a CNN to analyse photographic images to identify and classify mosquitoes by species and gender.

When adequate data are available, many methods, such as the Support Vector Machine (SVM) and Tangent Distance Classifiers (TDCs), can achieve good accuracy in automatic classification[42]. In a report by LeCun et al. on handwritten character recognition, more than 60,000 handwritten characters were used, resulting in a significantly reduced error rate[42]. Additionally, the ImageNet Large-Scale Visual Recognition Challenge (ILSVRC) uses approximately 1.2 million images for training, 50,000 for validation, and 150,000 images for testing[39].

In another application of a convolutional neural network for the identification of *Aedes* wings and larva, Lorenz et al.[26] used 17 species from the *Anopheles*, *Aedes* and *Culex* genera to test the hypothesis that classification based on wing shape characteristics using an Artificial Neural Network (ANN) was more accurate than traditional classification using discriminant analysis. The ANN was able to correctly classify species with greater accuracy than the conventional method using multivariate discriminant analysis, with an accuracy varying from 86% to 100%. However, this study focus on parts of the body of the mosquito and requires the use of laboratory equipment.

Another challenge in the use of convolutional neural networks for image recognition with a limited number of images is the risk of overfitting. Application of different sets of images during the training, validation and testing phases may reduce the risk of overfitting[39]. Additionally, the use of different, randomly selected images during the testing phase, as demonstrated in our study, is a promising method to avoid overfitting. Limitations due to dataset size and its balance between the classes may explain the low accuracy achieved for some species classified individually in our study. We firmly believe that increasing the dataset for all three phases (training, validation and testing) and the use of different neural network architectures will improve the precision of automatic classification.

Another possible way to increase the reliability of our neural network for the identification of mosquito images is to re-design the layers of the neural network to include additional morphological characteristics, such as wing shapes, palps and other features.

This preliminary study is an opportunity to construct a dense dataset of images of mosquitoes that can transmit diseases to humans, including *Anopheles* and other species among the 3,556 species of *Culicidae* that are currently recognized.

The final goal is to embed the model in a mobile APP that will allow for community participation and thereby facilitate efforts to control vector borne diseases.

## Conclusion

This work demonstrates that CNN's can be applied to the autonomous classification of mosquitoes to aid in the screening of possible vectors of arboviruses by health workers and other non-taxonomists. High accuracy of classification of mosquitoes using CNN's can be achieved using different neural networks. LeNet and AlexNet had inferior performances to that of GoogLeNet, which indicates that the use of more complex networks with more layers is necessary to improve accuracy. Taxonomic classification of insects can be performed automatically via the acquisition of image's features that differentiate one insect from another. Only by using both the genus and sex we achieved high accuracy in the identification of *Aedes* females.

Our results provide information that is fundamental to the automatic morphological classification of a species of interest applying CNN to classify the adult mosquito in the environment it lives. In the critical discussion, the published works develop mainly a laboratory tool. In the taxonomy of adult mosquitoes, an epidemiological classification has a different implication. The application method we propose will be a robust epidemiological instrument for the rapid identification of focus of mosquitoes allowing the community to be part of the control of vector-borne diseases.

Another essential contribution relies on the construction of a mosquito dataset. Much data is required to increase the reliability of any autonomous method to recognize objects. This study already provides more than 4,000 mosquito images, which is vital in creating a significant and robust dataset for later applications.

We hope that later, the model can be embedded in a mobile APP to allow for community participation and thereby facilitate efforts to control vector borne diseases. Indeed, this model can be used to improve vector control operations that are linked with fast and reliable identification of targeted species and to provide knowledge of their biology and ecology.
